# An efficient IoT-based crop damage prediction framework in smart agricultural systems

**DOI:** 10.1038/s41598-025-12921-8

**Published:** 2025-07-30

**Authors:** Nermeen Gamal Rezk, Abdel-Fattah Attia, Mohamed A. El-Rashidy, Ayman El-Sayed, Ezz El-Din Hemdan

**Affiliations:** 1 Department of Computer Science and Engineering, Faculty of Engineering, Kafrelsheikh University, Kafrelsheikh, Egypt; 2 Department of Computer Science and Engineering, Faculty of Electronic Engineering, Menoufia University, Menoufia, Egypt

**Keywords:** Crop damage, Missing data, Imputation technique, Machine learning, Ensemble learning, Prediction, Smart farming, Planetary science, Engineering

## Abstract

This paper introduces an efficient IoT-based framework for predicting crop damage within smart agricultural systems, focusing on the integration of Internet of Things (IoT) sensor data with advanced machine learning (ML) and ensemble learning (EL) techniques. The primary objective is to develop a reliable decision support system capable of forecasting crop health status classifying crops as healthy, pesticide-damaged, or affected by other stressors while addressing a critical challenge: the presence of missing data in real-time agricultural datasets. To overcome this limitation, the proposed approach incorporates robust data imputation strategies using both traditional ML methods and powerful EL models. Techniques such as K-Nearest Neighbors, linear regression, and ensemble-based imputers are evaluated for their effectiveness in reconstructing incomplete data. Furthermore, Bayesian Optimization is applied to fine-tune EL classifiers including XGBoost, CatBoost, and LightGBM (LGBM), enhancing their predictive performance. Extensive experiments demonstrate that XGBoost outperforms all other models, achieving an average sensitivity of 88.1%, accuracy of 89.56%, precision of 83.4%, and F1-score of 84.8%. CatBoost and LGBM also deliver competitive results, with CatBoost achieving 90.50% accuracy and LGBM reaching 90.23%. In addition, the imputation capability of the XGBoost model is validated through a low Mean Squared Error (MSE) of 0.0213 and a high R-squared (R^2^) value of 0.99, confirming its effectiveness for both prediction and data recovery tasks. The key contributions of this innovative work include the design of a low-cost, power-efficient, and scalable crop damage prediction system, the integration of real-time IoT data with optimized ensemble learning, and a comprehensive evaluation of imputation techniques to enhance model robustness. This framework is particularly suited for deployment in resource-constrained agricultural environments, advancing the field of smart farming through intelligent, data-driven solutions.

## Introduction

Agriculture, commonly known as farming, is the practice of cultivating crops and raising livestock, and it plays a vital role in the global economy. A significant portion of the world’s food supply and raw materials such as cotton and jute are derived from agricultural activities, serving as essential inputs for various industries and everyday products. Beyond food production, agriculture also supports the creation of numerous commercial goods. To ensure a successful harvest, appropriate resources must be provided from the moment seeds are sown, whether for nourishment or industrial use. Several key factors influence the quality of a harvest, including water availability, soil fertility, protection from pests, and the timely application of pesticides and other agricultural chemicals. While many of these factors are influenced by environmental conditions beyond human control, farmers can manage the type, amount, and frequency of pesticide application, making it a critical lever for improving crop yield and health.

The Internet of Things (IoT), combined with advanced data analytics techniques such as big data and data science, is increasingly shaping modern life by improving how individuals interact with their environment. In particular, IoT and data analytics are playing a vital role in the agro-industrial and environmental sectors, where they support the monitoring, diagnosis, and control of smart farming systems. These technologies offer essential insights to both farmers and consumers regarding the origin, quality, and attributes of agricultural products and systems^1^.

In recent years, a variety of advanced methods have been developed to tackle key challenges in smart farming, including disease detection, yield prediction, species identification, crop productivity enhancement, drought mitigation, and irrigation management. However, research in utilizing data analysis methods for improving decision support systems for farming data remains limited. Many existing approaches rely on traditional techniques without considering model performance, and some overlook data preprocessing in the initial stages. IoT offers solutions across various domains such as healthcare, security, smart homes, retail, smart cities, and agriculture. Its application in agriculture is particularly advantageous due to the continuous monitoring and control requirements in this field. In agriculture, IoT is deployed in micro-agriculture, livestock management, and greenhouse operations, each categorized into distinct control areas. These applications are monitored using Internet-connected sensors and devices, facilitated by wireless sensor networks (WSNs), enabling farmers to gather relevant data. Additionally, certain IoT-based devices remotely analyze and process data through cloud services, aiding researchers and agriculturists in making informed decisions.

Pesticides play a crucial role in protecting crops when applied in appropriate amounts. However, excessive use can be detrimental, potentially ruining the entire harvest and rendering the crops unfit for consumption. The level of pesticide application can be a determining factor in whether crops remain viable or suffer irreversible damage. Despite advancements in agricultural technology, conventional farming practices are still widely used around the world. These traditional methods, passed down through generations of experienced farmers, often rely on intuition rather than precision. As a result, they can be labor-intensive, time-consuming, and prone to inaccuracies, ultimately affecting productivity and sustainability.

Machine learning significantly enhances the effectiveness and simplicity of agricultural applications. Its core processes include data acquisition, modeling, and generalization. In many cases, machine learning algorithms are applied to solve complex problems where human expertise alone is insufficient. However, challenges such as network disconnections, hardware malfunctions, system breakdowns, detection failures, or connectivity issues during data transfer to web servers can disrupt data acquisition. These failures often result in data gaps by preventing the recording of key daily measurements. Consequently, second-order time-based variations may go unrecorded, leading to incomplete or imperfect datasets, a common issue in real-time data collection. As a result, values for some recorded variables may be partially or entirely missing. To address this, data imputation techniques serve as a practical solution. Imputation helps preserve the continuity of proprietary sensor and hardware data that exhibit temporal variation. This process involves modeling the distribution of each variable with missing values based on the patterns observed in the available data^2^.

The validity of imputation outcomes relies heavily on the rigor and appropriateness of the underlying modeling approach. Imputation should not be treated as a routine, one-click solution. In this study, various machine learning (ML) and ensemble learning (EL) techniques are explored for data imputation, including K-Nearest Neighbors (KNN) imputer, linear regression, and Extreme Gradient Boosted Decision Trees (XGBoost). Among these, XGBoost has demonstrated particular effectiveness due to its ability to handle missing values within the distance metric itself, reducing the need for explicitly defined imputation models. The proposed method estimates missing data using a supervised ML approach capable of adapting to new data based on learned patterns. Moreover, the use of advanced statistical simulation techniques has shown that EL models can more accurately reconstruct the true distribution of data compared to individual learners. In this research, ML and EL methods are employed to address missing data challenges and produce a complete dataset. This refined dataset is then used to train an EL-based classification model aimed at predicting the health status of crops, distinguishing between healthy crops, those damaged by pesticides, and those affected by other factors^3^.

The proposed crop damage prediction system was evaluated using a dataset reflecting outcomes from the harvest season. It outperformed traditional machine learning methods, achieving superior results in terms of accuracy, precision, sensitivity, and F-score. Additionally, the performance of the missing data imputation process was assessed using Mean Squared Error (MSE) and the coefficient of determination (R²)^4^. In conclusion, the key contributions of this paper are as follows:

- Advancing Smart Agriculture in Resource-Constrained Regions: This study aims to promote the advancement of smart agriculture in regions with limited resources by introducing a cost-effective IoT-based crop prediction system tailored for smart farming applications. The proposed system is designed to offer reliable support for accelerating cultivation processes, improving environmental sustainability throughout the entire production cycle, and enhancing the overall quality of agricultural output. Emphasis is also placed on achieving low power consumption, ergonomic design, and high operational efficiency, resulting in a user-friendly platform with the primary objective of accurately forecasting crop damage.
- Addressing Missing Data Challenges: A critical contribution of this work is its approach to handling missing data within real-time agricultural datasets. Machine Learning (ML) and Ensemble Learning (EL)-based imputation techniques are utilized to estimate and fill in missing values, ensuring data reliability and completeness. Techniques such as K-Nearest Neighbors (KNN), linear regression, and ensemble-based imputation algorithms were applied to handle incomplete data entries. A comparative analysis of these methods was conducted to evaluate how different imputation strategies affect the quality and distribution of the reconstructed data.
- Ensemble Learning for Crop Health Classification: The study employs ensemble learning techniques—including majority voting and weighted aggregation—to predict the outcome of the harvest, classifying crops into three categories: healthy (alive), damaged by pesticides, or damaged by other factors. The performance of the proposed decision support system was evaluated through extensive experiments, demonstrating its ability to accurately assess crop conditions. The system’s effectiveness in imputing missing values was also validated using statistical metrics.
- Bayesian Optimization of Ensemble Classifiers: To further enhance model performance, Bayesian Optimization was applied to fine-tune hyperparameters of key ensemble classifiers, including CatBoost, XGBoost, and LightGBM. The optimized models significantly outperformed traditional approaches such as linear regression:
  - XGBoost achieved a sensitivity of 88.1%, accuracy of 89.56%, precision of 83.4%, and F-score of 84.8%.
  - CatBoost yielded 88.1% sensitivity, 90.50% accuracy, 83.3% precision, and 84.6% F-score.
  - LightGBM recorded 86.3% sensitivity, 90.23% accuracy, 81.1% precision, and 83.1% F-score.

For evaluating missing data imputation, the XGBoost model demonstrated the best results with a Mean Squared Error (MSE) of 0.0213 and an R-squared (R²) value of 0.99, indicating excellent predictive accuracy and generalization ability.

The remainder of this paper is structured as follows: Sect. 2 presents a review of related work relevant to the study. Section 3 details the proposed methodology underlying the framework. Section 4 discusses the experimental study and analyzes the results obtained. Section 5 introduces the high-level architecture of the IoT-based smart agriculture system. Finally, Sect. 6 concludes the paper and outlines potential directions for future research in this innovative domain.

## Related work

Handling missing data is a critical challenge in data-driven modeling across various domains, particularly in agriculture, construction, weather forecasting, and traffic systems. Several recent studies have proposed diverse strategies to address this issue, incorporating both classical and modern machine learning (ML) techniques, including imputation methods and deep learning models. In the agricultural domain, the authors in^5^ proposed a deep learning-based system for predicting soil moisture using auxiliary features such as crop data, weather information, and irrigation metrics. The study also introduced a fog computing-based architecture to mitigate connectivity issues in farms and deployed the model on a single-board computer, showcasing the potential of real-time ML deployment. K-nearest neighbors (KNN) imputation was used to address missing values in the dataset, enabling efficient and accurate model performance.

In^6^, the authors evaluated various imputation techniques to enhance dataset completeness before predicting the compressive and tensile strength of concrete. The study employed several hyperparameter-optimized ML models, among which Extreme Gradient Boosting (XGBoost) yielded the highest accuracy when used with KNN imputation (K = 10), demonstrating the impact of appropriate imputation on model performance.

A related effort in^7^ focused on supervised ML for embedding reconstruction in meteorological data. Support Vector Regression (SVR) achieved an 80% improvement in filling data gaps compared to traditional embedding techniques, significantly benefiting weather prediction applications and improving data quality in the MAGDA’S-9 dataset. The study in^8^ systematically compared imputation methods for missing categorical data in supervised classification tasks. Using benchmark datasets with varying levels of missingness, the authors showed that imputation methods such as KNN significantly enhanced predictive accuracy, especially in the presence of data perturbations. The results were comparable to state-of-the-art classifiers on the Adult dataset.

In the context of time-series environmental data^9^, explored several statistical imputation techniques including Kalman smoothing, ARIMA, and multiple linear regression. These were applied to hourly temperature, humidity, and wind speed data across four locations in Western Australia. A five-fold cross-validation with 10% artificial missing data demonstrated the robustness of these models under random missingness conditions. Further improving on Kalman-based approaches^10^, proposed a Customized Kalman Filter (CKF) tailored for state-dependent noise in water distribution systems. CKF demonstrated superior performance in reconstructing missing values under noisy conditions, particularly for continuous remote monitoring applications.

In^11^, the authors introduced an enhanced ensemble learning strategy combining two General Regression Neural Networks (GRNNs) and a neural-like SGTM structure. This auxiliary structure improved the accuracy of weighted output aggregation, outperforming conventional ensemble methods. Complementary to this, Ref.^12^ presented a non-iterative neural approach for missing data imputation using a high-speed linear neural-like structure based on successive geometric transformation modeling. This method significantly reduced training time compared to traditional imputation algorithms, offering a computationally efficient alternative.

A novel deep learning-based approach for traffic data imputation was introduced in^13^, where the raw data were first transformed into spatial-temporal images. These were then processed using a convolutional neural network (CNN)-based context encoder to reconstruct missing sections. This method demonstrated strong performance in capturing spatial dependencies. Lastly, a comprehensive review in^14^ examined the handling of missing data across multiple studies. It revealed that most papers either did not report missing data handling strategies or relied heavily on complete-case analysis (CCA). Only a small fraction adopted advanced techniques such as multiple imputations or built-in strategies like surrogate splits during model development and validation. Also, some work recently used in crop damage detection^15,16^.

Table 1 presents a concise overview of recent studies addressing missing data challenges across various domains such as agriculture. It highlights the techniques employed that ranging from classical statistical methods to advanced machine learning and deep learning approaches and their respective contributions. This summary demonstrates the growing importance of robust imputation strategies in enhancing model accuracy and reliability in data-driven applications.

The proposed prediction framework in this study follows a structured and effective multi-step process. Initially, the datasets are preprocessed by addressing missing data using mode imputation, followed by the application of training, testing, and K-fold cross-validation techniques. To enhance data completeness, various imputation methods—ranging from traditional statistical techniques to modern machine learning approaches—are explored and implemented. Subsequently, a range of machine learning and ensemble learning classifiers are employed, including Random Forest Classifier (RFC), Extra Trees Classifier (ETC), Bagging Classifier (BC), Ridge Classifier (RC), Decision Tree Classifier (DTC), as well as CatBoost, XGBoost, and LightGBM classifiers. Among these, the top-performing classifiers are selected and further optimized using Bayesian Optimization to identify the most effective hyperparameter configurations. Ensemble strategies such as weighted and voting ensemble learning are then applied to boost prediction performance. The models are evaluated and compared using key performance metrics including accuracy, precision, recall, and F1-score. Overall, the study presents a smart, adaptable, and robust prediction process based on machine learning and ensemble techniques, with a specific focus on imputing missing values in agricultural datasets.

**Table 1:** Summary of related work.

Work	Technique	Key contribution
^5^	Deep learning + Fog computing + KNN	Soil moisture prediction with real-time deployment on SBC
^6^	XGBoost + KNN (K = 10)	Improved prediction of concrete strength with optimized imputation
^7^	SVR + Embedding reconstruction	80% improvement in meteorological data quality
^8^	KNN + Supervised learning	Enhanced accuracy for categorical data with varying missingness
^9^	Kalman, ARIMA, MLR	Robust imputation on temperature, humidity, and wind speed data
^10^	Customized Kalman Filter (CKF)	Effective under noisy conditions for remote monitoring
^11^	Ensemble: GRNN + SGTM	Improved accuracy in weighted aggregation of predictions
^12^	Neural-like SGTM	Faster training with non-iterative geometric transformation modeling
^13^	CNN + Context encoder	Spatial-temporal imputation of traffic data via image transformation

## Proposed framework

**Fig. 1:**
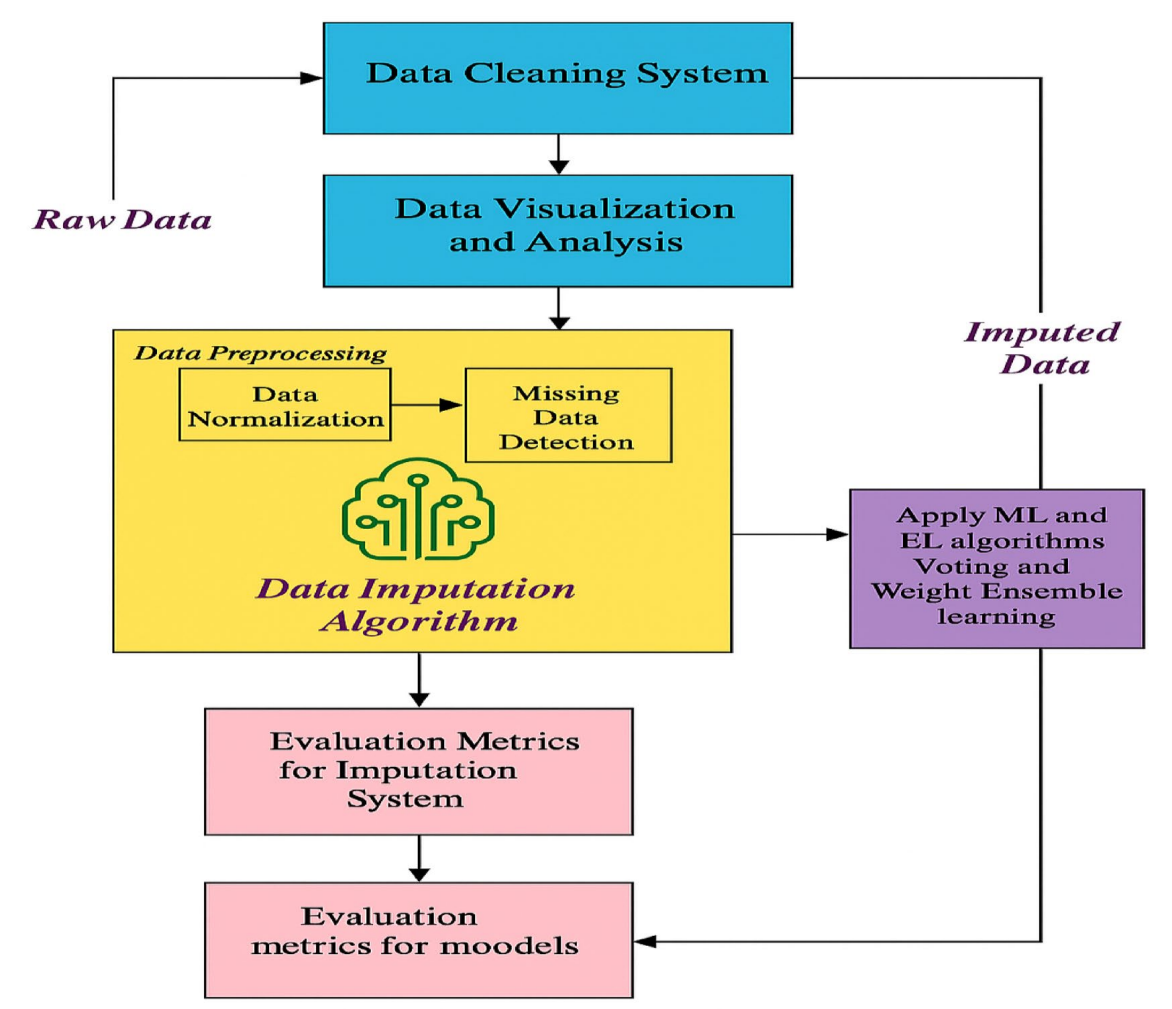
Proposed crop damage prediction framework.

This study presents an innovative and scalable framework for crop damage prediction in smart agriculture by addressing the critical issue of missing data using an integrated pipeline of data cleaning, imputation, and machine learning (ML) model optimization. Unlike previous works that focus solely on imputation algorithms, the proposed framework emphasizes the full system architecture, starting from raw sensor data processing to final model deployment. The methodological pipeline is illustrated in Fig. 1, and its core steps are described as follows:

- Step 1 (Data Cleaning System): The system begins with the ingestion of raw IoT-based agricultural data, which may contain noise, errors, or anomalies due to sensor failures or environmental factors. To ensure high-quality inputs for imputation, a data cleaning subsystem is triggered automatically when sufficient data is collected. This module employs threshold-based filtering to remove outliers and noisy data, which is essential to maintain the integrity of downstream imputation and modeling stages.
- Step 2 (Data Imputation System): The second stage addresses the core problem of missing data through the following components:
  - Data Preprocessing: Input features are normalized to a standard scale to handle variations in measurement units. Concurrently, the system identifies and flags missing values across the dataset for further processing.
  - Data Imputation Algorithm: Missing values are estimated using advanced imputation algorithms. The completed dataset is generated by filling each gap with appropriate values. This ensures that all subsequent ML and ensemble learning (EL) models are trained on consistent and complete data. The data cleaning step also ensures outlier removal, preventing skewed imputations caused by extreme values.
  - ML and EL Integration with Hyperparameter Tuning: Once the imputed dataset is ready, several ML and EL classifiers are applied, including: Random Forest (RFC), Extra Trees (ETC), Bagging Classifier (BC), Ridge Classifier (RC), Decision Tree (DTC), CatBoost, XGBoost, and LightGBM. Among these, CatBoost, XGBoost, and LightGBM demonstrated superior accuracy. To further improve performance, Bayesian Optimization was employed for hyperparameter tuning. This tuning process searches for the optimal model configurations to maximize prediction accuracy.
  - Hyperparameter Optimization: The optimized hyperparameters for top-performing algorithms are summarized in Table 2. These configurations define the architecture of each model before training. Once tuned, models are trained on the refined dataset and evaluated using validation metrics to determine the most effective algorithm for deployment.
- Step 3 (Evaluation Metrics for Imputation): Before applying ML models, the quality of imputation is assessed using Mean Squared Error (MSE) and Coefficient of Determination (R²). These metrics evaluate how well the imputed values approximate the missing data, ensuring the reliability of the reconstructed dataset.
- Step 4 (Ensemble Learning and Final Prediction): After validating the imputed dataset, all ML and EL classifiers are trained and evaluated. The framework further incorporates Voting and Weighted Ensemble Learning strategies to combine multiple model predictions, improving robustness and overall accuracy of crop damage classification.
- Step 5 (Evaluation Metrics for Prediction Models): The final performance of the prediction system is assessed using standard classification metrics such as Accuracy, Precision, Recall, and F1-Score. These metrics determine the model’s effectiveness in classifying crop health conditions, distinguishing between healthy crops, those damaged by pesticides, and those affected by other factors.

**Table 2:** Summary of all the hyperparameters used for the algorithms.

Algorithms	Hyperparameters used
CatBoost	iterations = 700, depth = 7, learning rate = 0.65, random strength = 6.998, bagging temperature = 0.77, border count = 135, l2_leaf_reg = 14
XGB	reg_alpha = 0, reg_lambda = 2, bagging_fraction = 0.999, min_split_gain = 0, min_child_samples = 10, subsample_freq = 3, subsample_for_bin = 50,000, n_estimators = 1000, max_depth = 6, min_child_weight = 108, subsample = 0.84, colsample_bytree = 0.8, verbose =−1
LGBM	n_estimators = 550, learning_rate = 0.03, min_child_samples = 40, random_state = 1, reg_alpha = 2, reg_lambda = 2, num_leaves = 36, max_depth = 37, scale_pos_weight = 2024, min_child_weight = 21, subsample = 0.85, colsample_bytree = 0.85

## Experimental study

This section offers the performance estimation and assessment of the prevailing suggested method.

### Experimental environment

The experiments are applied using machine learning and ensemble algorithms written in Python and running on Windows 10 with Intel(R) Core (TM) i9-7940X CPU @ 3.10 GHz processor and 64.0 GB RAM.

### Dataset

The suggested assay-based ML and EL models measure the outcome of the harvest season, i.e., whether the crop would be healthy (alive), damaged by pesticides, or damaged by other reasons using the agriculture data acquired from data sources^17^. Comprehensive information about this dataset like the number of attributes and the distribution of the dataset for training and testing based on 10-fold cross-validation is highlighted in Table 3.

**Table 3:** Comprehensive information of the dataset used.

The total number of samples in the AV agriculture datasets is 88,859.	
The distribution of the dataset for training and testing using a 10-fold cross-validation	
The missing values in the Number_Weeks_Used feature = 9000 features.	
Variable	Definition
ID	Unique ID
Estimated_Insects_Count	Estimated insect count per square meter
Crop_Type	Category of Crop (0,1)
Soil_Type	Category of Soil (0,1)
Pesticide_Use_Category	Type of pesticide uses (1- Never, 2-Previously Used, 3-Currently Using)
Number_Doses_Week	Number of doses per week
Number_Weeks_Used	Number of weeks used
Number_Weeks_Quit	Number of weeks quit
Season	Season Category (1,2,3)
Crop_Damage	Crop Damage Category (0 = alive, 1 = Damage due to other causes, 2 = Damage due to Pesticides) (label)

The agriculture datasets are treating missing values using (imputation using the simple importer (median), imputation using kNN, and imputation using Linear Regression and Extreme Gradient Boosted Decision Trees (XGBoost)) implemented into the machine learning and ensemble algorithm for the outcome of the harvest season and predict purpose. Then, using Bayesian optimized for the highest accuracy classifier to determine the hyperparameters for this classifier, and finally, apply the voting and weight classifiers.

### Performance metrics

The estimated capacity of the ML and EL techniques is assessed using both Mean Square Error (MSE) and Coefficient of determination (R2). The MSE evaluates the average Euclidean distance between the expected and true or calculated values and is displayed as^18,19^:

1 $$\:\text{M}\text{S}\text{E}=\frac{1}{n}\sum\:_{i=1}^{n}{({\hat{y}}_{i}-{y}_{i})}^{2}$$

where $\:{\hat{y}}_{i}$ and $\:{y}_{i}$ are the ith predicted output and the ith the true output, individually. MSE can evaluate the accuracy of the predicted values from each model, where lower MSE shows higher accuracy.

The coefficient of determination is exploited to determine the modification of the predicted values from the noted values. In this research, the Pearson correlation coefficient^18,20^ is employed to specify the accuracy of the predicted results. In the case of the collected data, the Pearson correlation coefficient can be calculated as follows:

2 $$\:{R}^{2}=\raisebox{1ex}{$\sum\:_{i=1}^{n}({y}_{p}\left(i\right)-{y}_{p}^{-}\left)\right({y}_{t}\left(i\right)-{y}_{t}^{-})$}\!\left/\:\!\raisebox{-1ex}{$\sqrt{\sum\:_{i=1}^{n}({y}_{p}\left(i\right)-{y}_{p}^{-})2}\:\:\sqrt{\sum\:_{i=1}^{n}({y}_{t}\left(i\right)-{y}_{t}^{-})2}$}\right.$$

Where $\:{y}_{p}\left(i\right)$ and $\:{y}_{t}\left(i\right)$ are the ith predicted output and the ith true output, respectively. in this paper, both MSE and R of the training data are exploited to get the best degree of complexity and performance for each ML and EL model.

The performance estimates of ML and EL models in a multiclass classification and prediction task are calculated by various statistical and mathematical models employed. These evaluation metrics such as accuracy, precision, f-score, and recall are exploited to equate the achievement of the suggested classifier to existing ones.

The observation is achieved by considering true negatives (TN), true positives (TP), false positives (FP), and false negatives (FN). The accuracy of the classification model on a defined test is the ratio of the test set that is accurately categorized by the classifier. Precision is the assessment of the correctness of positive labeled examples. Recall is the measure of fullness or accuracy of positive examples and how many examples of the positive class are labeled accurately. Accuracy, Precision, Sensitivity (Recall), and F_Score, are measured as per Eqs. (3)– (6) accordingly^21–25^.

3 $$\:\text{A}\text{c}\text{c}\text{u}\text{r}\text{a}\text{c}\text{y}\:=\frac{TP+TN}{TP+FP+FN+TN}$$

4 $$\:\text{P}\text{r}\text{e}\text{c}\text{i}\text{s}\text{i}\text{o}\text{n}\:=\frac{TP}{TP+FP}$$

5 $$\:\text{R}\text{e}\text{c}\text{a}\text{l}\text{l}\:=\frac{TP}{TP+FN}$$

6 $$\:\text{F}\_\text{S}\text{c}\text{o}\text{r}\text{e}\:=\frac{2*\left(R*P\right)}{P+R}$$

### Results analysis

Before conducting the experimental analysis, it was essential to properly prepare the data for machine learning and ensemble modeling. The real-world farming data collected from sensors exhibited non-uniform distribution, making it unsuitable for direct use in training and testing. To address this, input features were normalized, numerical columns were encoded, and categorical columns were processed using dummy encoding. These preprocessing steps ensured that the data was suitable for multiclass classification tasks.

**Fig. 2:**
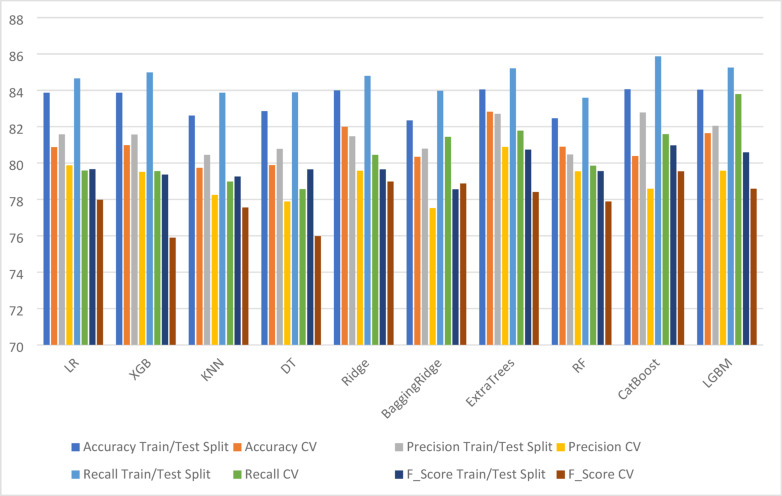
Comparative analysis between various ML algorithms for train/test split and cross validation (CV) for crop damage prediction.

Initially, we applied a train-test split (75% training, 25% testing) while handling missing values using mode imputation. We then used 10-fold cross-validation to further evaluate model performance. The results of these evaluations, including performance metrics for missing data imputation, are presented in Tables 4 and 5. Table 6 summarizes the outcomes of voting and weighted ensemble classifiers, while Table 7 shows the results of using a simple imputer for handling missing values. Figure 2 presents a comparative analysis of various machine learning algorithms under both train/test and cross-validation settings for crop damage prediction.

**Table 4:** Performance of ML models for missing data mode imputation using train and test split.

Algorithm	Performance metrics			
	Accuracy	Precision	Recall	F_Score
LR	83.864	81.58	84.65	79.66
XGB	83.863	81.57	84.98	79.36
KNN	82.611	80.45	83.87	79.26
DT	82.856	80.78	83.89	79.65
Ridge	83.996	81.47	84.79	79.65
Bagging ridge	82.340	80.79	83.97	78.56
Extra trees	84.047	82.71	85.21	80.74
RF	82.457	80.47	83.59	79.56
LGBM	84.04	82.05	85.25	80.59
Cat Boost	84.06	82.78	85.87	80.97

**Table 5:** Performance of the models for missing data mode imputation using cross-validation.

Algorithm	Performance metrics			
	Accuracy	Precision	Recall	F_Score
LR	80.874	79.88	79.59	77.98
XGB	80.985	79.51	79.56	75.89
KNN	79.741	78.25	78.98	77.56
DT	79.887	77.88	78.57	75.98
Ridge	81.997	79.58	80.45	78.98
Bagging ridge	80.347	77.52	81.44	78.87
Extra trees	82.821	80.89	81.78	78.41
RF	80.896	79.54	79.85	77.89
Cat boost	80.385	78.59	81.59	79.54
LGBM	81.640	79.58	83.79	78.59

**Table 6:** The performance of the voting and weight classified with different methods.

Algorithm	Accuracy	Imputation missing value using xgboost	Imputation missing value using linear Regression	Imputation missing value using KNN	Optimization using Bayesian optimization
Voting	89.9334	91.598	90.258	91.897	93.478
Weight	86.869	90.578	87.698	88.487	92.287

**Table 7:** The results of missing values imputation using simple imputer (SI).

Algorithm	Performance metrics			
	Accuracy	Precision	Recall	F_Score
LR	85.299	81.6	82.9	82.7
XGB	86.376	81.8	84.1	81.4
KNN	84.955	81.4	83.9	82.3
DT	84.996	82.7	82.8	82.7
Ridge	85.483	84.4	86.8	85.3
Bagging ridge	85.46	84.1	86.1	84.9
Extra trees	86.723	84.2	86.3	85.0
RF	84.439	84.4	86.8	85.2
Cat boost	86.58	81.9	84.1	81.2
LGBM	86.25	80.7	82.8	79.9

The evaluation technique to integrate missing data based on the pattern and the extent of data severity missing (from little to moderate to large amounts of data affected). Experiments on various standards of datasets imitating 10% of missing values artificially articulate the effect of missing patterns in the training and testing data sets. The imputation was chosen for the most occurrence of missing, this is usually experienced during missing data imputation of agriculture’s datasets disconnection data communication. The evaluation of the best structure to build the most optimized imputation of data is executed by trial and error by adapting the parameters of the supervised ML and EL models with their tuning values.

In the meantime, for KNN, tuning parameters to impute the missing values differ from 5 to 500 nearest neighbors. When KNN evaluates missing values depending on its neighbor, it potentially is prone to over-fitting and noise-sensitivity when K is too low or covers a large value of data points away from the neighbor, and while the impute data may occur to be bias-prone when it covers more instance space. Because this imputation technique contains an exploration of the full dataset to discover the KNN, it can be pricy and suffer from poor performance, especially for a huge dataset, and the results of the imputation of missing data using KNN imputation are shown in Table 8 while results for LR in Table 9. Between the stated supervised ML and EL models, KNN has been commonly chosen and performed in data imputation applications because of its ability to maintain the value of the missing data with the value of related cases (K-similarity of attributes) from the whole dataset, Missing data are measured by identifying a diversity of K-nearest neighbors, and then, meaning the non-missing values of its neighbors. The K value is determined stochastically depending on the Euclidean distance as in Eq. (7), by computing the square root of the sum of the difference between the calculated new value $\:{\hat{y}}_{k}$ and the novel value $\:{y}_{k}$. Based on the dataset size and ratio of missing values, the imputation process needs good tuning to avoid vulnerability to overfitting and sensitive data points^11,26–30^.

7 $$\:E\left(A,B\right)=\sqrt{\sum\:_{k=1}^{N}{({\hat{y}}_{k}-{y}_{k})}^{2}}$$

**Table 8:** The results of missing values imputation using KNN imputer.

Algorithm	Performance metrics			
	Accuracy	Precision	Recall	F_Score
LR	83.991	83.2	83.5	83.9
XGB	84.758	81.8	84.2	82.7
KNN	83.292	82.6	83.9	82.5
DT	80.80	80.4	80.3	80.4
Ridge	84.085	82.9	84.01	82.65
Bagging ridge	82.243	80.5	86.1	85.2
Extra trees	82.283	82.6	83.3	82.3
RF	83.014	81.9	83.7	82.6
Cat Boost	85.882	83.3	85.0	83.9
LGBM	85.578	82.9	85.7	80.8

On the other hand, XGBoost is dependent on a gradient-boosting approach, and it utilizes the ensemble of various weak models to reach the final predictions. Because each model in the XGBoost ensemble is established in the areas of data points, the algorithm process output training yields a rational XGBoost model which may Hence, using a cross-validation procedure and testing on the hidden test set, we can obtain a generalized model execute well on all the splits of the data. From Table 9, it can be noted that XGBoost has ascertained the underlying distribution and is supplying lower errors, and the performance is substantially enhanced after data imputations. It can be implicit that the selection of the best ML and EL approaches is based on the type of missing data imputation technique used. Considering that, XGBoost shows the highest performance, for the brevity of discussions, this paper essentially details the model performance for XGBoost (Table 10).

**Table 9:** The results of missing values imputation using linear regression (LR) imputer.

Algorithm	Performance metrics			
	Accuracy	Precision	Recall	F_Score
LR	85.515	83.4	85.1	85.4
XGB	84.395	82.9	84.8	84.1
KNN	83.984	79.5	80.9	83.8
DT	82.0118	83.9	83.3	82.6
Ridge	83.515	81.6	83.8	83.6
Bagging ridge	82.7400	80.2	82.2	82.8
Extra trees	86.439	82.5	86.1	86.9
RF	83.999	80.5	84.1	83.9
Cat boost	86.487	80.7	86.3	86.9
LGBM	86.445	81.2	86.2	82.9

**Table 10:** The results of missing values imputation using Xgboost imputer.

Algorithm	Performance metrics			
	Accuracy	Precision	Recall	F_Score
LR	87.515	83.9	85.6	86.5
XGB	87.395	81.4	86.7	83.5
KNN	85.984	76.8	81.3	78.3
DT	86.011	81.9	81.6	81.7
Ridge	87.515	83.7	86.9	85.6
Bagging ridge	86.7400	83.3	86.3	84.5
Extra trees	88.439	82.3	86.1	83.9
RF	86.999	82.5	86.7	84.2
Cat boost	88.487	81.3	86.7	83.5
LGBM	88.445	80.4	85.3	82.2

**Fig. 3:**
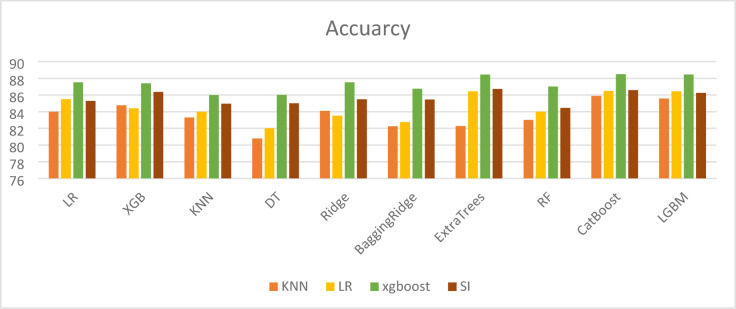
Accuracy results in different imputation algorithms.

**Fig. 4:**
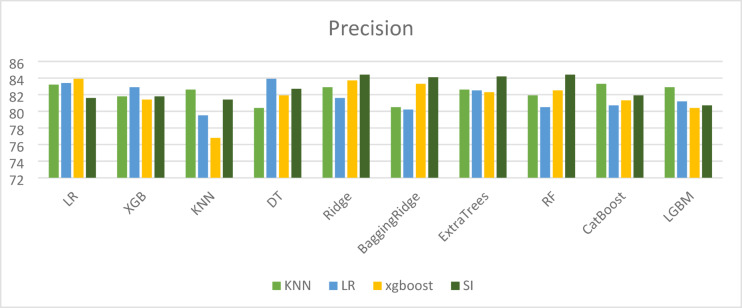
Precision results for different imputation algorithms.

**Fig. 5:**
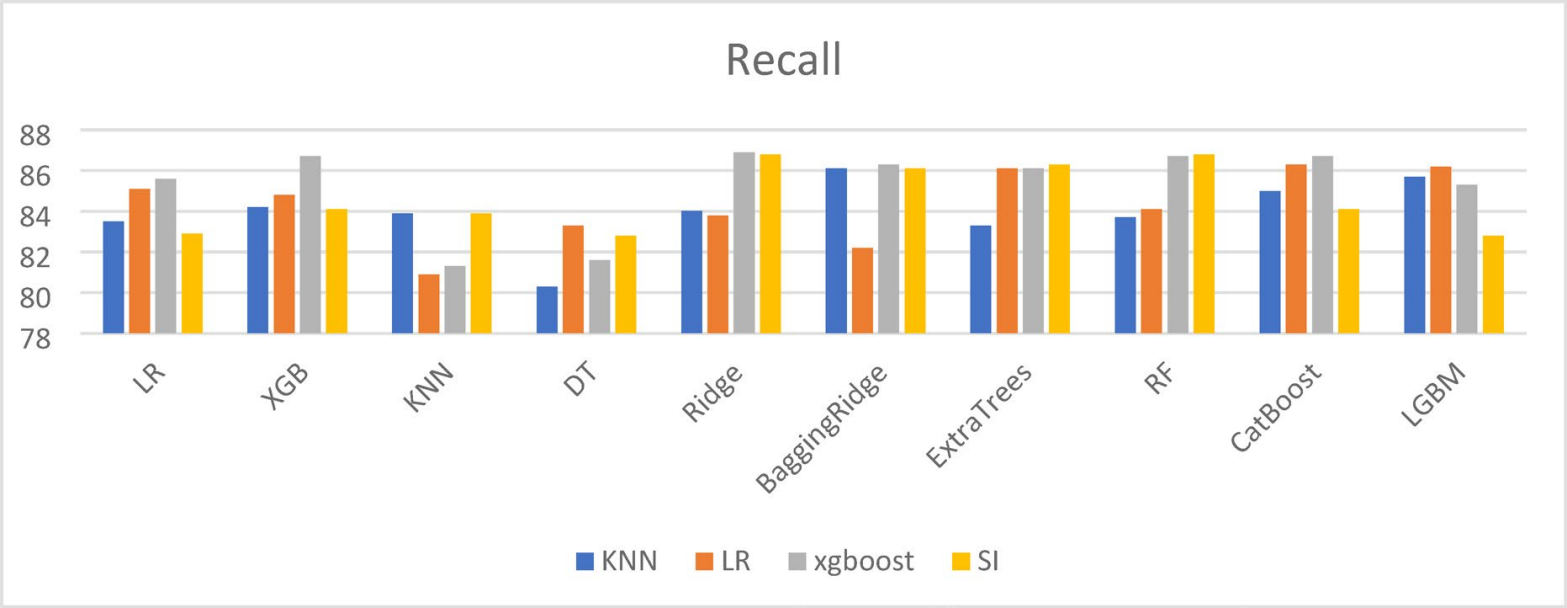
Recall results for different imputation algorithms.

**Fig. 6:**
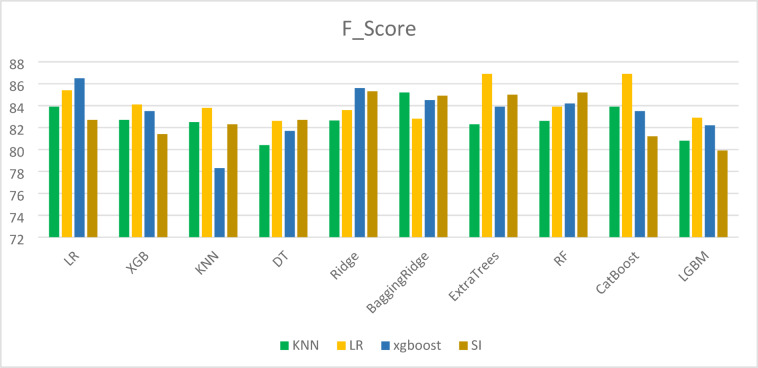
F1_Score results for different imputation algorithms.

To compare the performance of different machine learning techniques used for imputing missing values, both Mean Squared Error (MSE) and R-squared (R^2^) metrics were employed. While a high R^2^ value (close to 1) indicates that the model fits the known data well, it does not always guarantee accurate imputation. This is evident in the case of the simple imputer, which achieved an R^2^ of 0.96 but was associated with a relatively high MSE of 0.1682, highlighting inconsistencies in prediction accuracy. Therefore, reliable imputation should be assessed using both high R^2^ and low MSE values. As shown in Table 11, the XGBoost model outperformed other methods by achieving the lowest MSE and highest R^2^, indicating superior imputation accuracy. Visual comparisons of the results across different imputation methods are provided in Figs. 3, 4, 5 and 6.

**Table 11:** Performance comparison for missing value handling.

Algorithms	Performance metrics	
	MSE	*R* ^2^
Simple imputer	0.1682	0.96
KNN	0.0569	0.99
XGboost	0.0213	0.99
Linear regression	0.0483	0.98

In conclusion, this study establishes the effectiveness of supervised machine learning (ML) mechanisms and ensemble learning (EL) techniques for imputing missing data in the context of smart farming applications, particularly in predicting crop damage outcomes during the harvest season. Various ML models, including XGBoost, Linear Regression, KNN, and simple imputer, were explored. The dataset used in this study not only pertains to the harvest season outcomes (healthy, damaged by pesticides, or damaged by other reasons) but also presents a significant challenge with a substantial amount of missing data.

To address the complexities associated with missing data, multiple strategies were employed to build the presence of data gaps, enabling further agricultural event processing and subsequent analysis. The dataset exhibited variations in predicted tracking capability and performance metrics, evaluated through MSE, R2, accuracy, precision, recall, and F-score, depending on the imputation models and hyperparameter settings. Notably, the Extreme Gradient Boosted Decision Trees (XGBoost) technique, with optimal hyperparameters, demonstrated superior performance in imputing missing data, achieving low values of MSE (0.0213) and high R2 (0.99) for up to a 10% missingness ratio.

The suitability of the selected data imputation method was found to be influenced by factors such as the data pattern, missingness mechanism, data type, and the ratio of missing values—all of which impact performance estimation. This study highlights that machine learning and ensemble learning techniques outperformed traditional methods, with ensemble models showing particularly strong results compared to individual machine learning algorithms and linear regression.

Lastly, Table 12 presents a benchmarking comparison between the proposed model and recent studies. It highlights that while methods like DNN, IML, and statistical models show strengths in accuracy and interpretability, they often face limitations such as high computational cost, sensitivity to outliers, or complexity. The proposed system stands out for combining interpretable ML and ensemble learning (EL) with Bayesian Optimization, achieving high imputation accuracy. It effectively handles missing data through a hybrid ML-EL imputation approach, outperforming traditional techniques.

**Table 12:** Comparison between the proposed model with the recent work.

References	Method	Merit	Demerit	Handling missing values
^5^	Deep Neural Networks (DNN)	High accuracy in complex data patterns; suitable for large datasets.	Computationally expensive; requires large training data.	DNN-based imputation.
^6^	Interpretable Machine Learning (IML)	Improves prediction accuracy; provides interpretable results.	May struggle with high-dimensional data.	Missing data imputation via IML.
^7^	Supervised Machine Learning & Statistical Models	Effective for ground electromagnetism data combines ML and statistical robustness.	Sensitive to outliers; statistical assumptions may not always hold.	Regression-based imputation, Expectation-Maximization (EM).
^9^	High-resolution temporal imputation	Preserves temporal patterns; suitable for climate data.	May not generalize well to non-temporal datasets.	Time-series interpolation, Kalman filtering.
^11^	Improved GRNN-SGTM Ensemble	High imputation accuracy; robust to noise.	Complex implementation; requires parameter tuning.	Hybrid GRNN (General Regression Neural Network) and SGTM (Successive Geometric Transformation Model).
Proposed System	Interpretable IML and EL	High imputation accuracy and Applying Bayesian Optimization for CatBoost, XGBoost, and LightGBM.	Requires extensive hyperparameter tuning and computational resources for ensemble learning models.	Missing data imputation via Hybrid ML and EL

## High-level proposed IoT-based crop damage prediction system

**Fig. 7:**
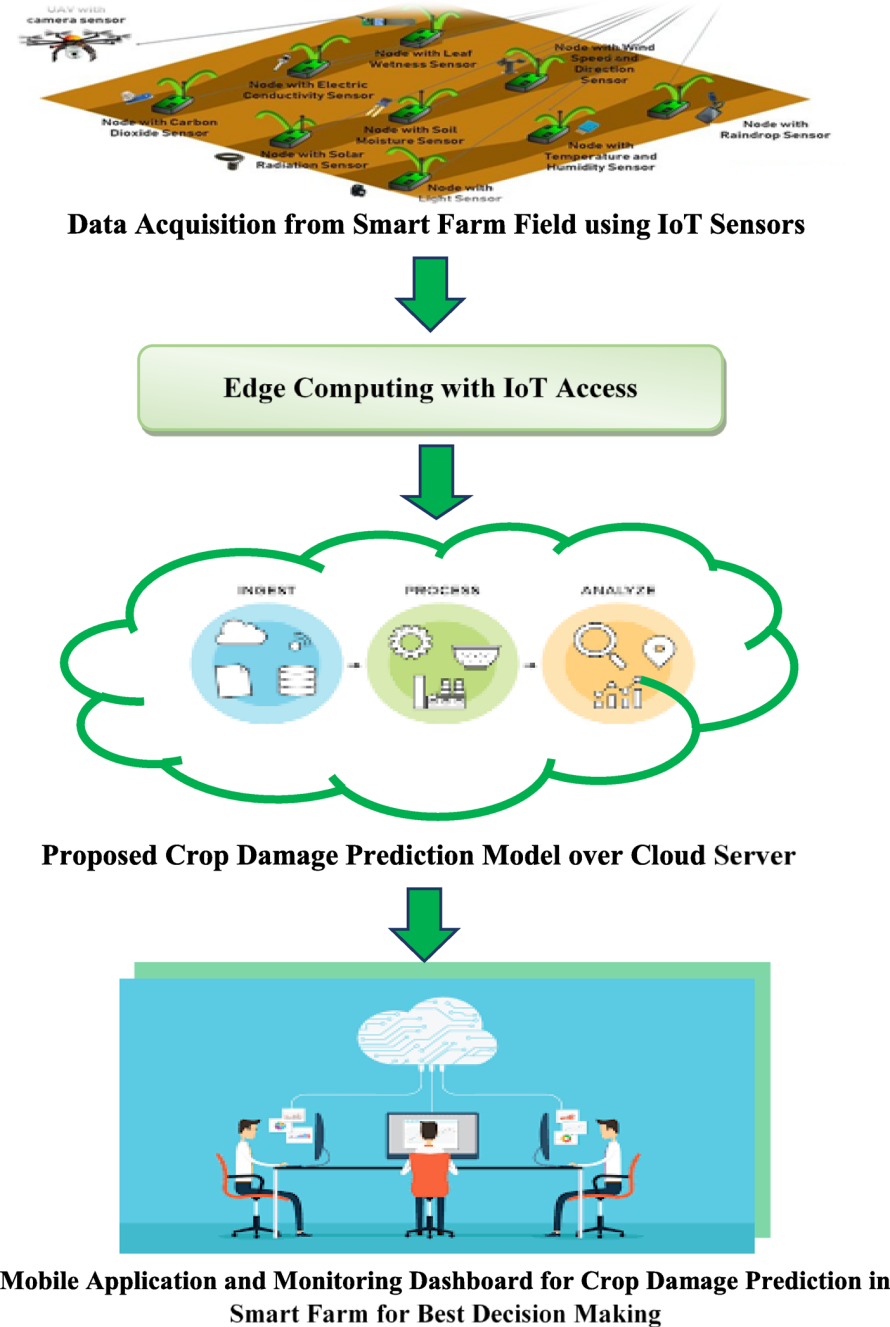
Acclaimed IoT-based crop damage prediction system in smart agriculture applications.

Figure 7 illustrates the proposed IoT system for crop damage forecasting. Within this system, IoT-based sensors are deployed to collect farming data within a smart farmhouse environment. Subsequently, the collected farm data are transmitted to an IoT-based gateway, such as Raspberry Pi. This gateway serves as an intermediary between the sensors and the analytics server hosted in the cloud, facilitating crop damage prediction for informed decision-making in smart farming systems. The controller periodically transmits the gathered data to the respective channels using an IoT communication protocol such as CoAP or MQTT.

Finally, mobile or web applications can interface with cloud-based analytics servers to obtain crop damage forecasting decisions using the proposed hybrid model depicted in Fig. 1. The prediction outcomes are then relayed to the management team within the smart farm, enabling them to take appropriate actions.

In summary, taking an overview of the proposed IoT-based Crop Damage Prediction System that clarified in Fig. 7 which can be as follows:

1. IoT-based farming system’s constituents:
  IoT-based sensors: Deployed within a smart farmhouse environment to collect farming data.
  IoT gateway: Acts as an intermediary between sensors and the cloud-hosted analytics server. (e.g., Raspberry Pi)
  Analytics server: Hosted in the cloud, responsible for crop damage prediction.
  Controller: Manages data transmission from sensors to the gateway using IoT communication protocols (e.g., CoAP, MQTT).
  Mobile/web applications: Interfaces with cloud-based analytics servers to access crop damage forecasting decisions.
2. Data flow for crop damage prediction:
  Farming data collected by sensors is transmitted to the IoT gateway.
  The gateway facilitates the transmission of data to the cloud-hosted analytics server.
  The Analytics server processes data for crop damage prediction.
  Prediction outcomes are relayed to management teams via mobile/web applications.
3. Decision-making for smart farming:
  Management teams use the prediction outcomes to make informed decisions regarding crop management and damage mitigation strategies within the smart farm.

## Conclusion and future scope

The findings of this study offer practical insights into handling missing data in IoT-based smart farming systems, supporting more reliable decision-making. Among the evaluated models, XGBoost achieved the highest performance with sensitivity, accuracy, precision, and F1-score of 88.1%, 89.56%, 83.4%, and 84.8%, respectively. CatBoost and LGBM also performed competitively. XGBoost further demonstrated strong imputation accuracy with a low MSE of 0.0213 and a high R² of 0.99. For future work, we aim to develop a more generalized and efficient model by applying advanced feature engineering techniques to improve input selection and reduce training time. We also plan to explore ensemble methods like SVR and GRNN-SGTM with optimized hyperparameters to enhance predictive performance and computational efficiency in smart agriculture applications.

- Generalized Model Improvement: Explore and enhance a more generalized imputation model by incorporating advanced feature engineering techniques. This can involve refining input feature selections, eliminating unnecessary features, and optimizing model training efficiency without compromising predictive accuracy.
- Advanced Prediction Models and Deep Learning: Future work will focus on implementing advanced ensemble methods such as ensemble Support Vector Regression (SVR) and GRNN-SGTM, as well as incorporating deep learning models to capture more complex patterns in agricultural data. This combined approach aims to improve prediction accuracy and robustness while optimizing hyperparameters to reduce computational time and enhance model efficiency.
- Integration of Real-Time Data: Incorporate real-time data sources and explore the integration of dynamic data into the prediction framework. This can enhance the model’s adaptability to changing agricultural conditions, ensuring that predictions remain relevant and accurate over time.
- User-Friendly Decision Support System: Develop a user-friendly decision support system based on the crop damage prediction framework. Empower farmers and stakeholders with accessible tools that provide actionable insights, recommendations, and visualizations to aid decision-making in smart farming practices.
- Validation and Field Testing: Perform comprehensive validation and real-world field testing to evaluate the practical effectiveness of the proposed framework. Collaborating with agricultural experts and practitioners will help assess model predictions under real farming conditions and enable continuous refinement based on field feedback.
- Future Work with Diverse Datasets: Extend the study by incorporating larger and more diverse datasets across different crops, regions, and environmental conditions. This will enhance the model’s generalizability and ensure its robustness in varied agricultural scenarios.

By addressing these future points, the efficiency and applicability of the crop damage prediction framework in smart farming systems can be further improved, contributing to sustainable and data-driven agricultural practices.

## Data Availability

The datasets used and/or analysed during the current study available from the corresponding author on reasonable request.
